# SARS-CoV-2 Omicron variants: burden of disease, impact on vaccine effectiveness and need for variant-adapted vaccines

**DOI:** 10.3389/fimmu.2023.1130539

**Published:** 2023-05-23

**Authors:** Shanti Pather, Shabir A. Madhi, Benjamin J. Cowling, Paul Moss, Jeremy P. Kamil, Sandra Ciesek, Alexander Muik, Özlem Türeci

**Affiliations:** ^1^ BioNTech, Mainz, Germany; ^2^ South African Medical Research Council Vaccines and Infectious Diseases Analytics Research Unit, Faculty of Health Sciences, University of the Witwatersrand, Johannesburg, South Africa; ^3^ School of Public Health, The University of Hong Kong, Hong Kong, Hong Kong SAR, China; ^4^ Institute of Immunology and Immunotherapy, University of Birmingham, Birmingham, United Kingdom; ^5^ Department of Microbiology and Immunology, Louisiana State University Health Sciences Center Shreveport, Shreveport, LA, United States; ^6^ Institute for Medical Virology, University Hospital Frankfurt, Goethe University Frankfurt, Frankfurt, Germany

**Keywords:** sub-lineage, BA.1, vaccine, disease burden, Omicron

## Abstract

The highly transmissible Omicron (B.1.1.529) variant of severe acute respiratory syndrome coronavirus 2 (SARS-CoV-2) was first detected in late 2021. Initial Omicron waves were primarily made up of sub-lineages BA.1 and/or BA.2, BA.4, and BA.5 subsequently became dominant in mid-2022, and several descendants of these sub-lineages have since emerged. Omicron infections have generally caused less severe disease on average than those caused by earlier variants of concern in healthy adult populations, at least, in part, due to increased population immunity. Nevertheless, healthcare systems in many countries, particularly those with low population immunity, have been overwhelmed by unprecedented surges in disease prevalence during Omicron waves. Pediatric admissions were also higher during Omicron waves compared with waves of previous variants of concern. All Omicron sub-lineages exhibit partial escape from wild-type (Wuhan-Hu 1) spike-based vaccine-elicited neutralizing antibodies, with sub-lineages with more enhanced immuno-evasive properties emerging over time. Evaluating vaccine effectiveness (VE) against Omicron sub-lineages has become challenging against a complex background of varying vaccine coverage, vaccine platforms, prior infection rates, and hybrid immunity. Original messenger RNA vaccine booster doses substantially improved VE against BA.1 or BA.2 symptomatic disease. However, protection against symptomatic disease waned, with reductions detected from 2 months after booster administration. While original vaccine-elicited CD8^+^ and CD4^+^ T-cell responses cross-recognize Omicron sub-lineages, thereby retaining protection against severe outcomes, variant-adapted vaccines are required to expand the breadth of B-cell responses and improve durability of protection. Variant-adapted vaccines were rolled out in late 2022 to increase overall protection against symptomatic and severe infections caused by Omicron sub-lineages and antigenically aligned variants with enhanced immune escape mechanisms.

## Introduction

The Omicron variant (Pango lineage B.1.1.529, GISAID clade GR/484A) of severe acute respiratory syndrome coronavirus 2 (SARS-CoV-2) was first detected in November 2021 (1, 2). This first Omicron sub-lineage, now known as BA.1, contains numerous mutations, particularly in the spike protein, resulting in enhanced transmissibility, partial escape from previously established neutralizing antibodies to wild-type (Wuhan-Hu-1) SARS-CoV-2, and risk of re-infection (3–5). A sister lineage, BA.2, emerged soon after. Omicron was not the first variant of concern (VOC) to exhibit neutralizing antibody evasion; the Beta variant in particular exhibited partial resistance to neutralization by antibodies elicited by initial pandemic waves and first-generation vaccines (6). Unlike the Beta variant, which circulated for only a short time in a limited number of countries, the high reproductive rate of BA.1 and BA.2 allowed these variants to rapidly disseminate around the world. Although primary infection caused by BA.1 or BA.2 appeared to be generally less severe than previous VOCs, with case numbers doubling far quicker than previous waves, the emergence of these variants has been considered a turning point in the pandemic.

Owing to the large number of individuals infected with BA.1 or BA.2 and the high transmissibility of these variants, several sub-lineages of Omicron have since emerged (2, 7). This led to further waves of infection with sub-lineages BA.4 and BA.5 in many countries; BA.5 descendent lineages are now dominant globally (8). In this review, we describe the characteristics of Omicron B.1.1.529 and its sub-lineages, the associated burden of disease, and the impact of this group of variants on vaccine effectiveness (VE) in the context of evolving infection- and vaccine-induced population immunity. This review was written at a time when the BA.4, BA.5, and BA.2.75 sub-lineages were dominant in most regions and bivalent variant-adapted vaccines were being rolled out. Newer sub-lineages have since emerged and key data on these sub-lineages were added during peer review. The initial literature search was performed on June 20, 2022, with additional searches conducted on a case-by-case basis, owing to the rapidly evolving nature of this topic.

## Characteristics of Omicron B.1.1.529 and sub-lineages

### Phylogenetics and impact of genetic alterations

**Figure 1:**
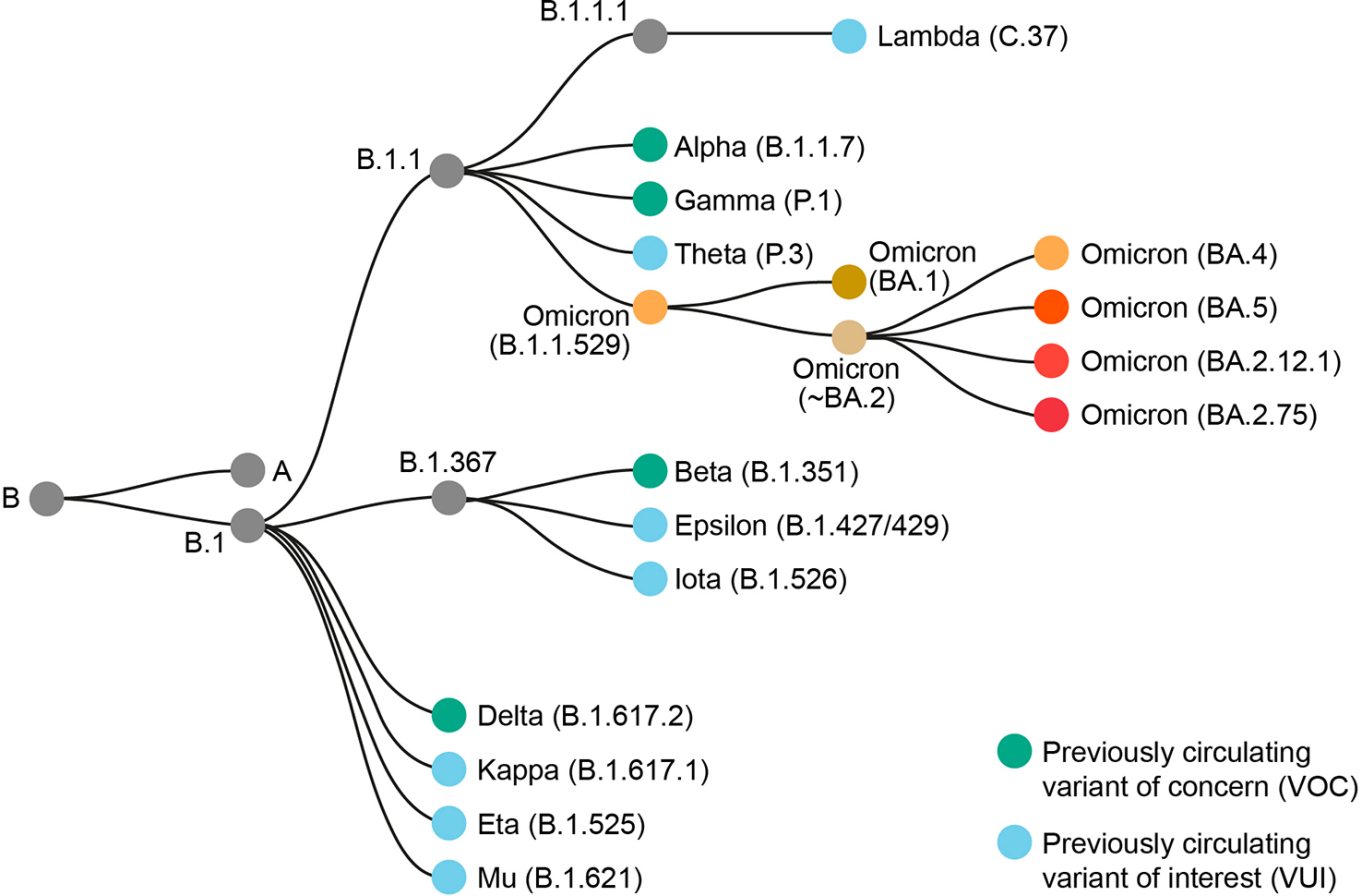
Phylogenetic relationship of Omicron B.1.1.529 and sub-lineages to other SARS-CoV-2 variants. Adapted from Nextstrain (7).

There has been significant intra-variant evolution since BA.1 was first detected in November 2021. BA.1 was supplanted by other Omicron sub-lineages, as was BA.3. Most initial BA.2 sub-lineages were supplanted by descendant sub-lineages such as BA.2.75, BA.4, and BA.5. Omicron sub-lineages currently being monitored by the World Health Organization are BA.5 sub-lineages BQ.1 (including descendent lineage BQ.1.1) and BF.7, BA.2 descendent lineages BA.2.75 and CH.1.1, and XBB (including descendent lineage XBB.1.5) (2). XBB is a recombinant of BA.2.10.1 and BA.2.75 (2). The term ‘Omicron’ encompasses BA.1, BA.2, BA.3, and all subsequent B.1.1.529 sub-lineages (2). The Omicron group of variants forms a new phylogenetically distinct clade that is not directly descended from other SARS-CoV-2 VOCs (9) (**Figure 1**). Of all the previous VOCs, BA.1 is most closely related to the Alpha and Gamma variants, underscoring those ancestors of the Omicron variant sub-lineages that remained in circulation during the Delta variant wave (9).

Key substitutions and deletions in the genomes of Omicron sub-lineages with known functions are shown in **Table 1**. A large number of these are linked to evasion of receptor-binding domain (RBD)- or N-terminal domain (NTD)-directed neutralizing antibodies (10, 12, 15, 16, 28), or enhanced binding to the angiotensin-converting enzyme 2 receptor (14, 17–19, 22). However, results from at least two studies in mice suggest that mutations affecting viral genes outside of S play critical roles in the reduced pathogenicity of Omicron lineages relative to earlier VOCs such as Delta or the ‘ancestral’ 2019 Wuhan lineage (29, 30). Alterations in non-S genes have been linked to changes in protein stability (24), increased host immune suppression (25), and enhanced sub-genomic RNA expression leading to increased viral load (26, 27, 31).

**Table 1:** Key genetic substitutions and deletions of Omicron BA.1 and sub-lineages BA.2, BA.4, BA.5, and BA.2.12.1.

Mutation	Omicron sub-lineage (7)	Possible importance
S gene/spike protein		
del69–70	BA.1, BA.4, BA.5	Resistance to neutralizing antibodies/infectivity (10, 11)
G142D	BA.2, BA.4, BA.5, BA.2.1.12	Resistance to NTD neutralizing antibodies (12)
del143–145	BA.1	Resistance to NTD neutralizing antibodies (12)
R346K	Only found in a subset of sequences (2)	Resistance to neutralizing antibodies (12)
S371L	BA.1	Resistance to RBD neutralizing antibodies (all classes) (12)
N440K	BA.1, BA.2, BA.4, BA.5, BA.2.1.12	Resistance to neutralizing antibodies (12), increased fusogenicity (13)
G446S	BA.1	Resistance to neutralizing antibodies (12)
L452R	BA.4, BA.5	Increased binding to ACE2 (14)
L452Q	BA.2.12.1	
F486V	BA.4, BA.5	Broadly neutralizing antibody evasion (15)
E484A	BA.1, BA.2, BA.4, BA.5, BA.2.12.1	Neutralizing antibody evasion (16)
Q493R	BA.1, BA.2, BA.2.12.1 (Reverted to R493Q in BA.4 and BA.5)	Increased binding to ACE2 (17), neutralizing antibody evasion (12)
N501Y	BA.1, BA.2, BA.4, BA.5, BA.2.12.1	Increased binding to ACE2 (18)
H655Y	BA.1, BA.2, BA.4, BA.5, BA.2.12.1	Increased binding to ACE2/transmissibility (19); enhancement of endosomal entry (20, 21)
P681H	BA.1, BA.2, BA.4, BA.5, BA.2.12.1	Transmissibility (22); enhancement of endosomal entry (21)
N969K	BA.1, BA.2, BA.4, BA.5, BA.2.12.1	Reduced fusogenicity to S2 domain (23)
ORF1a/NSP6		
del3674–3676	BA.1	Protein stability (24)
del3675–3677	BA.2, BA.4, BA.5, BA.2.12.1	
ORF9b		
del27–29	BA.1, BA.2, BA.4, BA.5, BA.2.12.1	Suppression of immune response (25)
N gene/nucleocapsid		
R203K G204R	BA.1, BA.2, BA.4, BA.5, BA.2.12.1	Subgenomic RNA expression, increased viral load (26, 27)

ACE2, angiotensin-converting enzyme 2; del, deletion; NSP, non-structural protein; NTD, N-terminal domain; ORF, open reading frame; RBD, receptor-binding domain; RNA, ribonucleic acid.

**Figure 2:**
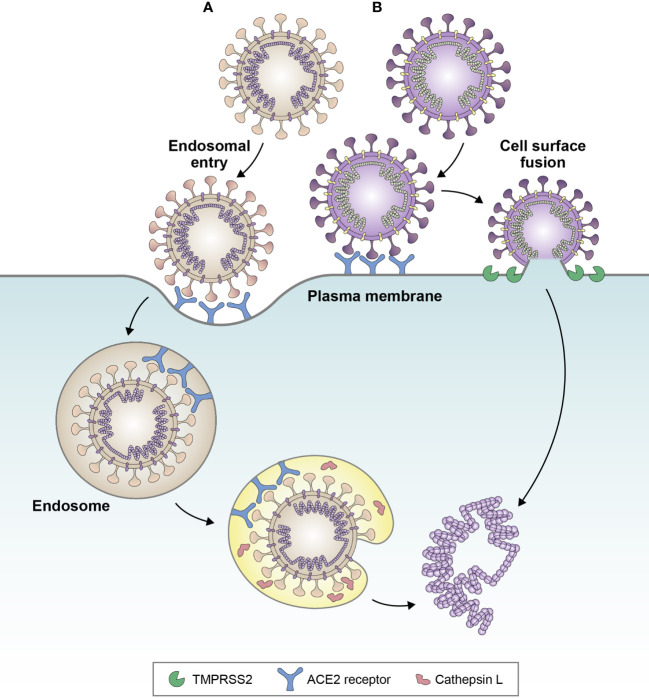
Favored cell entry pathways of **(A)** BA.1 and **(B)** Delta variant. Delta favors cell surface fusion, whereas BA.1 favors endosomal entry. Evidence suggests that BA.4 and BA.5 sub-lineages may be partially reverting back towards cell surface fusion, due to increased fusogenicity compared with BA.1. Adapted from Tang et al. *Antiviral Res* (2020);178:104792 (32).

Importantly, alterations to the S gene of the BA.1 and BA.2 sub-lineages have led to a fundamental change in the entry route of Omicron into host cells. While previous VOCs, such as Delta, were able to enter host cells via surface fusion following transmembrane protease serine 2-mediated proteolysis, BA.1 and BA.2 exhibit reduced fusogenicity, proteolysis, and syncytia formation, and have therefore switched entry pathway preference towards cathepsin-dependent fusion within the endosome (**Figure 2**) (20, 23, 28, 33). Enhanced endosomal entry and reduced fusogenicity have occurred as a result of alterations at the S1/S2 cleavage site and in the S2 domain, leading to reduced furin pre-processing, and retaining stabilization of the spike protein in the ‘closed’ conformation (20, 21, 28), which makes the RBD less accessible to neutralizing antibodies. As such, BA.1 and BA.2 have altered cell tropism compared with previous VOCs, favoring the upper respiratory tract (33).

Later Omicron sub-lineages, BA.4 and BA.5, have been shown to exhibit increased fusogenicity compared with BA.2, which has been attributed to the L452R and N440K substitutions (13, 34). Sub-lineages BA.4.6, BQ.1, and BQ.1.1 exhibit enhanced fusogenicity compared with BA.4 and BA.5 (13). The loss, and subsequent reattainment, of fusogenicity by Omicron sub-lineages is consistent with the Canyon Hypothesis (35), in which sustained viral transmission in seropositive populations leads to the emergence of variants with closed RBD configurations (such as BA.1/BA.2), reducing susceptibility to neutralizing antibodies. Circulation in populations with decreasing neutralizing antibody levels then leads to the emergence of variants with viral entry proteins favoring open configurations for more efficient cell entry (36). The F486V mutation of BA.4 and BA.5 has been shown to provide resistance to class 1 and class 2 RBD antibodies (15) and may have facilitated the move back towards the open configuration. In line with this, infection experiments in a guinea pig model have linked increased fusogenicity of BA.4 and BA.5 versus BA.2 with increased pathogenicity (37).

Antigenic drift has occurred rapidly with SARS-CoV-2 since the pandemic began and has led to a quick succession of changes in viral fitness. The emergence of recombinant variants, such as XBB and its descendants, demonstrates that antigenic shift may also occur, resulting in viruses that are antigenically distant from previously circulating variants and that have more evolved immuno-evasive mechanisms (38). The high frequency of changes in the genome of SARS-CoV-2 and the possibility of antigenic shift suggest that regular updates to coronavirus disease 2019 (COVID-19) vaccines may be required in the future, in order to provide protection against emerging antigenically distinct variants.

### Immune evasion

When compared with previous VOCs, neutralizing antibodies induced by original COVID-19 vaccines to BA.1 are reduced, as is memory B-cell recognition (39). BA.2 evades infection- and original vaccine-induced neutralizing antibodies with a comparable level of efficiency to BA.1 (40). Both BA.1 and BA.2 have been shown to evade neutralizing antibodies elicited by a primary series of messenger RNA (mRNA; mRNA-1273 or BNT162b2), vector-based (Ad26.COV2, Sputnik V, or ChAdOx1 nCoV-19), subunit (NVX-CoV2373), and inactivated (BBIBP-CorV) vaccines (41), although some activity is retained from mRNA or ChAdOx1 nCoV-19 vaccination. Original vaccine booster doses can restore neutralizing antibody activity against these Omicron sub-lineages to an extent, depending on the vaccine platforms used. Homologous vaccination with three doses of original mRNA vaccine (mRNA-1273 or BNT162b2), or heterologous vaccination with a vector-based vaccine (Ad26.COV2.S, ChAdOx1 nCoV-19, or Sputnik V) followed by an original mRNA booster, improves neutralization potency against BA.1 and BA.2 (41–43). In individuals vaccinated with a primary series of inactivated vaccine, a homologous booster enhances neutralizing antibody responses to BA.1, but to a lesser extent than heterologous mRNA vaccine boosters (44–47). Neutralizing antibody responses to BA.1 and BA.2 after original mRNA vaccine booster dosing have been shown to decline over time, reaching low levels at 4–6 months post-booster (48, 49). The rate of neutralizing antibody decay seems to be similar to that of wild-type SARS-CoV-2 (48). T-cell responses elicited by original mRNA vaccines against BA.1 are generally maintained for longer than humoral responses, suggesting that protection against severe disease may be preserved despite neutralizing antibody decay (50); however, T-cell responses may begin to wane from 6 months after infection (51).

Evidence suggests that hybrid immunity (vaccine-induced immunity in individuals who have also been infected) after a primary series of inactivated vaccine plus single original mRNA vaccine booster may generate higher neutralizing antibody responses to BA.2 than a second booster of either original mRNA or inactivated vaccine in individuals without previous infection (49).

Later Omicron sub-lineages, such as BA.4 and BA.5, seem to evade cross-protection from BA.1 infection. In individuals vaccinated with original vaccines, breakthrough BA.1 disease results in strong neutralizing antibody activity against BA.1 (52), reactivating memory B cells (53), and a less substantial increase in neutralizing antibody titers against BA.4 and BA.5 (52). Neutralizing antibody levels against BA.4 and BA.5 in non-vaccinated individuals with prior BA.1 infection are lower than those in individuals vaccinated with original vaccines and with BA.1 breakthrough infection (54). Neutralizing antibody titers are lower against BA.4, BA.5, and BA.2.12.1 than against BA.1 in individuals with prior BA.1 infection (17, 34, 54, 55). Exposure to BA.2 results in greater neutralizing activity against BA.4 and BA.5 than exposure to BA.1, driven by antibodies targeting the NTD of the spike protein (56). This is likely due to the fact that BA.2 is more closely phylogenetically related to BA.4 and BA.5 than BA.1 is (56, 57). BA.4 and BA.5 are antigenically distinct and are as distant from BA.1 as BA.1 is from the wild-type virus (38).

BA.4, BA.5, and BA.2.12.1 also escape original vaccine-elicited neutralizing antibodies to a greater extent than BA.1 or BA.2 (17, 34, 54, 58). BA.4 and BA.5 escape sera from individuals who received a primary series and booster of original mRNA vaccine to a greater extent than BA.2.12.1 (4.2-fold vs. 1.8-fold greater than BA.2 in one study (15) and by a factor of 3.3 vs. 2.2 compared with BA.1 in another (58)). However, individuals vaccinated with three doses of original mRNA vaccine with BA.4/BA.5 breakthrough infections have been shown to exhibit broad and robust neutralizing activity against BA.1, BA.2, BA.2.12.1, BA.4, and BA.5 (57). Nonetheless, BA.2.75.2, BQ.1, BQ.1.1, XBB, XBB.1, and CH.1.1 have been shown to exhibit lower neutralization sensitivity than BA.4/BA.5, indicating further neutralization escape with newly emerging sub-lineages (13, 38, 59–62). This is due to the phylogenetic distance between the XBB and BQ sub-lineages and BA.4/5 (38).

Owing to an extensive number of substitutions in the S gene relative to the ‘ancestral’ 2019 virus, Omicron variants are epistatically poised for escape from a variety of different antibodies (63). Notably, the efficacy of several monoclonal antibody therapies against Omicron sub-lineages BA.2.12.1, BA.4, and BA.5, as well as BQ and XBB sub-lineages, has been shown to be limited, although small-molecule antivirals retain activity (38, 64). There is also evidence that Omicron sub-lineages, as well as other VOCs, have developed relative resistance to interferons, affecting the host innate immune response (65).

Overall, neutralizing activity conferred by original COVID-19 vaccines is limited against newly emerging, antigenically distant sub-lineages; a pattern similar to that observed with influenza. The current trajectory suggests that SARS-CoV-2 will continue to evolve towards more immune-evasive variants, further affecting the effectiveness of vaccines and therapeutics. This further supports the need for regular updates to COVID-19 vaccine composition and booster vaccination to restore protection against circulating variants.

### Transmissibility and re-infection

The transmissibility of BA.1 is clearly greater than that of previous VOCs, as demonstrated by its rapid global spread. BA.2 has been shown to be more transmissible than BA.1 (66), possibly attributable to an increased viral load in the upper pharynx (67). BA.4, BA.5, and BA.2.12.1 may be more transmissible than BA.2 (17, 68); however, owing to their increased fusogenicity, they also spread more efficiently in human lung cells, which suggests that they may be more likely to manifest as a lower respiratory tract infection (37). XBB.1.5 has an additional growth advantage over other Omicron sub-lineages, with a doubling time of 9 days (69). This is due to a higher angiotensin-converting enzyme 2 binding affinity compared with earlier variants, such as XBB.1 and BQ.1.1, which is conferred by the S486P mutation (70).

The risk of re-infection is increased with Omicron sub-lineages versus other VOCs (71). In Qatar, prior infection with pre-Omicron variants has been shown to provide less protection against symptomatic BA.1 infection than against symptomatic disease caused by other VOCs (56% vs. 85–92%, respectively) (72). In a study in Scotland, the proportion of BA.1 cases that were possible re-infections was >10 times greater than the proportion of Delta cases that were re-infections (7.6% vs. 0.7%, respectively) (73). This may be due to the fact that Omicron variants have become dominant, with prolonged circulation and resurgences compared with Delta and other previous VOCs. Nevertheless, prior infection with pre-Omicron variants does seem to provide a similar level of protection against severe, critical, or fatal disease due to re-infection with BA.1 versus other VOCs (72). This may also be partially due to the greater propensity of BA.1 for upper respiratory tract infection compared with other VOCs.

Immunity from prior infection can have a large role in protection against Omicron sub-lineages in countries with low vaccination rates. For example, in South Africa, high seroprevalence of SARS-CoV-2 immunoglobulin G after the Delta wave led to an apparent decoupling of infection from hospitalizations during the first Omicron wave (74). However, as new antigenically distinct sub-lineages with greater capacity for immune evasion emerge, the benefits of prior infection may be reduced, especially with waning of immunity over time. For example, in Qatar, effectiveness of pre-Omicron infection against BA.2.75 was 6%, and effectiveness of BA.1 or BA.2 infection against BA.2.75 was 50% (75). Protection conferred by prior infection against more antigenically distant and immune-evasive BQ sub-lineages is further reduced, as demonstrated by the increased re-infection risk with XBB sub-lineages (76).

### Prevalence, severity, and burden of disease

The high transmissibility of Omicron sub-lineages and relative neutralizing antibody evasiveness have led to a rapid and substantial increase in disease prevalence, in terms of number of infections, compared with the pre-Omicron period, which has been sustained through continued replacement by emerging sub-lineages. Globally, the BA.1 wave began in November/December 2021 and peaked in January/February 2022 (77). BA.1 was then largely replaced by BA.2, and BA.2 was subsequently displaced by BA.4 and BA.5 around August 2022; the BA.2 descendent sub-lineage BA.2.75 also began to circulate at this time (77). Newer sub-lineages with enhanced immuno-evasive properties, including BQ.1.1, CH.1.1, and XBB.1.5, are now circulating in multiple countries (8). As Omicron sub-lineages are now dominant, it is reasonably likely that future VOCs will evolve from recent Omicron sub-lineages. It is also still possible that new VOCs will arise from non-dominant circulating viruses or earlier branches of the SARS-CoV-2 phylogenetic tree, as was observed with Omicron (9).

In many countries, the severity of infections in Omicron BA.1 waves has been milder than that of previous VOCs in terms of risk of hospitalization (73, 78–80), need for mechanical ventilation, and death (81, 82). These data must be interpreted in the context of continually evolving population immunity derived from infection and/or vaccination. Similarly, it is difficult to compare the clinical severity of each Omicron sub-lineage on this background of increasing and geographically variable population immunity. The current evidence does not indicate any significant change in disease severity associated with BA.2.12.1, BA.4, or BA.5 compared with BA.2, indeed, the reduced severity of BA.1 and BA.2 versus previous VOCs has persisted with BA.4 and BA.5 (83, 84), although BA.4 and BA.5 did not circulate at a time of significant circulation of other respiratory viruses in the Northern Hemisphere. There are no data to suggest an increase in disease severity with currently circulating sub-lineages, such as BQ.1.1 or XBB.1.5 (76, 85). There have been considerable regional differences in the severity of Omicron infections, likely linked to differences in population immunity. In South Africa, where infection-induced immunity was high and vaccine coverage was low, the BA.4/BA.5 wave has resulted in less severe infections than the BA.1 wave (86), with limited impact on healthcare services (74). In the United Kingdom, where vaccination coverage was high, the rise in prevalence during the BA.1 and BA.2 waves was associated with increases in hospitalizations and deaths, but at lower levels than previous waves (87). The impact has been greater in countries where vaccination rates or prior infection rates are low. The BA.2.2 wave in Hong Kong, where both vaccination coverage in older adults and prior infection rates were low, resulted in more than 1 million cases and close to 10,000 deaths (88, 89), and the BA.5 wave in New Zealand, where infection-induced immunity was low, led to a peak 7-day rolling average of 25 deaths per day, placing additional strain on the capacity of hospitals already overburdened with high caseloads (90). Estimates suggest that in China, which previously had very low rates of prior infection, up to 248 million people (18% of the population) may have been infected during the first 20 days of December 2022 (91).

Elderly, immunocompromised, comorbid, and unvaccinated populations remain at high risk of severe disease resulting from Omicron infection (78, 92). In addition, pediatric infections seem to be more frequent with Omicron sub-lineages versus earlier variants. Pediatric admissions were higher during the BA.1 wave than with previous waves (93–95), and SARS-CoV-2 seroprevalence in pediatric populations substantially increased, reaching 68–77% across age groups in the United States by February 2022 (96). In some countries, this has translated to increased severity in children, whereas in others, severity in children has been similar to, or lower than, previous waves. In Hong Kong, 2% of unvaccinated children hospitalized with BA.2 required admission to pediatric intensive care and two deaths were recorded (97). In the United Kingdom, the risk of hospital admission with Omicron BA.1 infection in children <10 years of age was comparable to that of Delta (98). In South Africa, although SARS-CoV-2 seropositivity in children <12 years of age reached 84% after the BA.1 wave (86), and the incidence of pediatric hospitalizations was similar to previous waves, mortality was lower (74). These differences are likely due to geographical variations in vaccination coverage and prior infection in pediatric populations. Pediatric admissions have also increased during circulation of the BQ.1.1 sub-lineage in the United States, with many States reporting that 90% of pediatric beds are occupied. However, other seasonal respiratory viruses such as respiratory syncytial virus and influenza have also been circulating in children during this period (99, 100).

Some evidence suggests a change in the rate of COVID-19 complications in pediatric populations with Omicron sub-lineages compared with previous VOCs. BA.1 has been associated with a significant increase in upper airway infection in children (4.1% during an Omicron-dominant period vs. 1.5% in the pre-Omicron period in the United States; p < 0.001) (101), although this may be partially attributable to increased testing rates. Of patients with croup at a US hospital, 48% were infected with SARS-CoV-2 during the BA.1 wave compared with only 3% during the Delta wave (102). Conversely, the relative risk of multisystem inflammatory syndrome after BA.1 infection in unvaccinated children in Denmark was significantly reduced compared with the Delta wave (0.12; 95% confidence interval [CI]: 0.06–0.23; p < 0.001) (103).

An increase in infections in pregnant women has also been observed during the BA.1 wave compared with Delta and pre-Delta periods (median 138 vs. 14 and 17 cases per week, respectively, in a study in the United States), with the majority occurring in unvaccinated individuals (104). The majority of these cases were non-severe, with an odds ratio for severe or critical illness versus the pre-Delta period of 0.20 (95% CI: 0.05–0.83). Severity of BA.1 may have been mitigated by evolving population immunity.

Omicron has also resulted in numerous healthcare worker (HCW) absences, further exacerbating pressure on healthcare systems. In England, approximately 40% of HCW absences in the last week of January 2022 (a BA.1-dominant period) were due to COVID-19 (105). Persistent symptoms are common in people recovering from COVID-19 and can hinder their ability to work (106). Thus, the presence of persistent symptoms may result in increased and/or prolonged HCW absences. Omicron outbreaks also increased stress among HCWs, potentially leading to further absences. In a survey of HCWs in Saudi Arabia, uncertainties around Omicron during the BA.1 wave were significantly correlated with stress, leading to reduced resilience and ability to cope (107).

Measures that countries have taken to support healthcare systems during Omicron waves include postponing elective surgeries to free up staff and beds, deploying military personnel to support hospitals, and recalling retired HCWs (108).

In summary, the emergence of the highly transmissible Omicron variant and its subsequent sub-lineages resulted in an increase in hospitalizations and HCW absenteeism, placing additional burden on already overstretched healthcare systems. The current evidence demonstrates that populations such as the elderly, people with co-morbidities, and pregnant women, remain at high risk of severe outcomes of Omicron infection, including hospitalization and death. This supports the need for continued booster vaccinations in these populations as SARS-CoV-2 continues to evolve.

## Vaccine effectiveness and development needs

### Immunogenicity and effectiveness of original vaccines

As described earlier, Omicron sub-lineages exhibit partial escape from humoral immunity induced by current vaccines. In vaccinated individuals, the acute B-cell response to BA.1 breakthrough is mediated by vaccine-induced B-cell clones with a bias toward recognition of ancestral SARS-CoV-2 (52, 53). BA.1 breakthrough infection induces a shift towards the formation of memory B cells against epitopes that are broadly conserved across variants, with a robust recall response (52, 53). When compared with BA.1 convalescent patients, vaccinated individuals have been reported to have lower levels of BA.1-reactive B cells, as well as lower levels of neutralizing antibodies in bronchoalveolar lavage fluid (109). This may suggest that infection induces a greater mucosal immune response than vaccination. In line with these data, hybrid immunity has been shown to provide greater protection against symptomatic BA.1 or BA.2 disease than vaccination alone (110). In Qatar, effectiveness of three doses of BNT162b2 against symptomatic BA.2 infection in individuals with no prior infection at a median of 43 days post-booster was 52%, while effectiveness in individuals with a prior infection was 77% (110). Hybrid immunity from infection and booster vaccination seems to confer the greatest neutralization capacity (111), with broader activity and cross-reactive antibody affinity maturation against BA.1 and BA.2 versus infection-naïve booster-vaccinated individuals (112). Individuals vaccinated with three doses of mRNA vaccine with BA.4/BA.5 breakthrough infections exhibit broad and robust neutralizing activity against BA.1, BA.2, BA.2.12.1, BA.4, and BA.5 (57).

Although Omicron sub-lineages evade vaccine-elicited humoral responses to varying degrees (41, 54, 58), cell-mediated immunity to Omicron sub-lineages remains robust in vaccinated individuals. T-cell responses induced by different vaccine platforms, including mRNA (mRNA-1273 and BNT162b2), vector-based (Ad26.COV2.S), and subunit vaccines (NVX-CoV2373), cross-recognize variants from Alpha to BA.1 (39, 113–115). In vaccinees from the United States and South Africa, CD8^+^ T-cell responses to the spike protein of BA.1 induced by BNT162b2 or Ad26.COV2.S were >76% and CD4^+^ T-cell responses were >70% that of T-cell responses to the wild-type spike protein (114, 115). Some variation has been observed by vaccine platform; for example, in vaccinated individuals from Hong Kong, T-cell responses to BA.1 were higher in those who received two doses of BNT162b2 than in those who received two doses of the inactivated vaccine CoronaVac (CD8^+^: 81.8% vs. 71.4%; CD4^+^: 96.7% vs. 82.1%, respectively) (116). Conservation of the cell-mediated immune response in the lung may be associated with prevention of severe disease (109). This may partially explain why, despite the greater neutralization capacity of hybrid immunity versus vaccine-induced immunity, limited differences in VE against severe outcomes were observed between individuals with three mRNA vaccine doses with or without prior infection (110).

In VE studies across different settings, a booster dose of mRNA vaccine resulted in transient improved VE versus symptomatic disease during BA.1- or BA.2-dominant periods (117–122), but VE waned quickly over time. In England, VE against symptomatic BA.1 disease after a primary series of BNT162b2, mRNA-1273, or ChAdOx1 nCoV-19 followed by an mRNA booster had decreased at 5–9 weeks post-booster and further declined at >10 weeks (117). VE against symptomatic BA.2 disease decreased from 74% at 7 days post-booster to 44% at ≥15 weeks (119). In Qatar, effectiveness of a booster dose of BNT162b2 against symptomatic Omicron infection (any sub-lineage during a period of BA.1 and BA.2 dominance) decreased from 56% at 2–3 weeks post-booster to 22% at ≥14 weeks. Similarly, effectiveness of an mRNA-1273 booster decreased from 54% at 2–3 weeks post-booster to 35% at ≥6 weeks (121). In the United States, VE of mRNA-1273 against BA.1 infection decreased from 71.6% after 14–60 days post-booster to 47.4% after >60 days (118), and VE of BNT162b2 against BA.1 infection in HCWs decreased from 75% within 8 weeks to 55% at >16 weeks (120). However, VE against severe outcomes of BA.1, BA.2, or BA.4/5 infection has been very high following an mRNA booster dose (119, 122–127). In the United States, VE of three doses of mRNA vaccine against invasive mechanical ventilation or in-hospital death during the BA.1 wave was 94% (124). In Canada, VE against severe outcomes of BA.1 infection was 95% ≥7 days post-mRNA booster (122). In England, VE against BA.2 hospitalization post-mRNA booster peaked at 89% (119). A booster dose was also associated with a reduction in the risk of hospitalization and death due to BA.5 infection in Portugal (128). In children 5–11 years of age, effectiveness of a third dose of BNT162b2 against Omicron-related emergency department or urgent care encounters (any sub-lineage) was 77% after a median of 43 days post-booster (129). In the United States, effectiveness of three doses of BNT162b2 against BA.4/BA.5-related emergency department visits was 71% <3 months post-booster (127), and, in South Africa, effectiveness of three doses of BNT162b2 against hospitalization during the BA.4/BA.5 wave was 69% at 1−2 months post-booster (126).

VE against severe outcomes of BA.1, BA.2, and BA.4/BA.5 infection also wanes from 3–4 months after administration of the booster dose, albeit to a lesser extent than VE against symptomatic disease (94, 119, 126, 127, 130, 131). In some countries, a fourth vaccine dose has been administered to the elderly and/or HCWs, in order to provide additional protection against Omicron sub-lineages for these high-risk populations. In Israel, during a BA.1-dominant period, the rate of confirmed infection was reduced 2-fold and the rate of severe illness by 3.5-fold at 4 weeks after a fourth dose of BNT162b2 in adults >60 years of age compared with those who had only received three doses (132). Breakthrough COVID-19 infection rates in HCWs in Israel who received a fourth dose were lower than in those who received three doses during the BA.1 wave (7% vs. 20%, respectively) (133). In Ontario, Canada, VE of mRNA vaccines against symptomatic infection and severe outcomes in long-term care residents ≥60 years of age during a BA.1-dominant period was 69% and 86%, respectively, ≥7 days after the fourth dose (134). Given that booster doses provide additional protection against severe outcomes of Omicron infection, including hospitalization and death, continued vaccination of high-risk populations is likely.

Duration of neutralizing antibody protection against infection is a function both of sustained activity over time and variant sensitivity to neutralizing antibody activity. Based on the observed waning of VE against infection after booster administration (119, 130), and the fact that neutralizing antibody titers against Omicron sub-lineages are lower than those against prior VOCs after a booster dose (5, 43, 135), boosters against prior circulating VOCs may not address the complex needs posed by waning immune responses and new variants with enhanced transmissibility or pathogenicity. Therefore, adaptation of vaccines to include new VOCs, thus allowing increased duration and breadth of protection against infection, is highly desirable.

### Variant-adapted vaccines to address Omicron and future variants

Variant-adapted vaccines are vaccines that have been updated to provide improved immune responses against a specific variant or variants (136). As well as addressing the specific variant/variants, these vaccines have the potential to increase the breadth of primary neutralizing antibody responses against other Omicron sub-lineages and prior VOCs when compared with the original vaccines (136, 137), through the formation of memory B cells against the new variant and robust recall of old memory B responses (52). Preservation and expansion of the T-cell response may potentially provide more durable protection against severe disease and deaths over time (138).

The need for variant-adapted vaccines was recognized after the emergence of BA.1, and mRNA vaccine manufacturers subsequently initiated the development of BA.1-adapted vaccines (139, 140). By the time data on the BA.1-adapted vaccines had been generated, sub-lineages BA.4 and BA.5 had increased in prevalence and were expected to become dominant. Data from BA.1 and BA.2 convalescent serum from triple mRNA-vaccinated individuals showed low neutralizing antibody titers against BA.4 and BA.5, suggesting that BA.1-adapted vaccines may not provide high levels of protection against BA.4/BA.5 infection (56). However, data from BA.4/BA.5-infected and triple mRNA-vaccinated individuals revealed robust neutralization of BA.4/BA.5 and all previously circulating Omicron sub-lineages (57). Manufacturers therefore also initiated development of BA.4/BA.5-adapted mRNA vaccines. Based on preliminary data on a prototype BA.4/BA.5-adapted vaccine, in July 2022, the United States Food and Drug Administration (FDA) recommended that modified vaccines should have a BA.4/BA.5 spike protein component in addition to the existing composition to create a bivalent booster for use in both adult and pediatric populations in Q3/Q4 2022 (141, 142).

Both the FDA and European Medicines Agency (EMA) have provided guidance on regulatory requirements for variant-adapted vaccines, stating that effectiveness can be established on the basis of immunogenicity bridging studies demonstrating superiority of the neutralizing antibody response elicited by the adapted vaccine compared with the prototype vaccine in terms of geometric mean titer ratios (143, 144). Based on such data, the EMA has granted full marketing authorization for a bivalent original BNT162b2/Omicron BA.1 vaccine in individuals ≥12 years of age, and for a bivalent original BNT162b2/Omicron BA.4-BA.5 vaccine in individuals 5–11 years of age (pediatric formulation) and ≥12 years of age (140). Bivalent mRNA-1273/Omicron BA.1 and mRNA-1273/Omicron BA.4-5 vaccines have received EMA approval for use in individuals ≥12 years of age (139). The FDA has granted Emergency Use Authorization for original BNT162b2/Omicron BA.4-BA.5 vaccines in individuals ≥5 years of age and mRNA-1273/Omicron BA.4-5 vaccines in individuals ≥6 years of age (145).

Early clinical data show that the bivalent BA.4-5 BNT162b2 vaccine induces greater pan-Omicron neutralizing activity and substantially higher neutralizing antibody titers against BA.4/BA.5 compared with the original vaccine in adults ≥55 years of age (136). The bivalent BA.4-5 BNT162b2 vaccine elicits T-cell responses to emerging immuno-evasive variants, such as XBB.1.5, albeit slightly reduced when compared with responses to the wild-type virus (146). Real-world data will further strengthen evidence for the effectiveness of these variant-adapted vaccines. Emerging data from the US Centers for Disease Control and Prevention (CDC) indicate a 2.4-times-lower risk of death due to COVID-19 in vaccinated individuals who received an Omicron BA.4-5 vaccine booster compared with those who did not (147). Although evidence suggests that the XBB.1.5 sub-lineage can partially escape neutralizing antibodies elicited by bivalent BA.4-5 boosters, VE estimates are similar to those against BA.5: CDC estimates suggest effectiveness against symptomatic infection in adults of 37–52% against BA.5 and 43–39% against XBB/XBB.1.5 at ≥2 weeks post-booster (61, 148).

Although the bivalent vaccines provide protection against currently circulating variants, given the continuing emergence of more antigenically distant sub-lineages and the increasing resistance of these sub-lineages to neutralizing antibodies, it is likely that further variant-adapted vaccines will be required as SARS-CoV-2 continues to evolve. In line with this, the Vaccines and Related Biological Products Advisory Committee anticipate conducting vaccine composition evaluations for COVID-19 vaccines at least annually, with a potential variant change to be selected in May 2023 (149). Bivalent vaccines may be implemented in vaccine-naïve populations as well as being administered as boosters, based on EMA Emergency Task Force guidance (150). Specific areas that still require global decision-making for future variant-adapted vaccines include the rationale for taking a monovalent versus a bivalent approach, and the rationale for requiring an updated vaccine, as well as the specific variant that should be included.

### Next-generation vaccines

Ultimately, the value in developing a variant-adapted vaccine will always be mitigated by the time taken to detect the variant and develop and approve the vaccine (151), as well as the challenges to improving durability of protection during the frequent evolution of SARS-CoV-2. Several approaches to the development of novel vaccines that elicit a broader immune response and increased durability of protection against current and future variants are being researched (151–156), including candidates with enhanced prefusion spike proteins to improve magnitude and breadth of immune response (156), and candidates that target other non-spike antigens (153, 155). T-cell-enhanced vaccines composed of T-cell antigens encoding non-spike proteins that are conserved across variants are being evaluated and may have potential to expand protection against severe disease (157). Novel adjuvants designed to trigger specific components of innate immunity are being explored (158), and intranasal vaccines to elicit an enhanced immune response at the nasal mucosa are also in development (159).

Pan-sarbecovirus vaccines that provide broad and durable protection against all members of the sarbecovirus subgenus (i.e. SARS-CoV-2, SARS-CoV-1, and various non-human coronaviruses) are particularly desirable, and several approaches to this are under investigation (153, 155). Several mosaic nanoparticle vaccines, composed of numerous copies of the RBD or prefusion spike protein from SARS-CoV-2 and related viruses, such as Middle East Respiratory Syndrome coronavirus or coronaviruses circulating in bats, are in early development. A mosaic nanoparticle vaccine containing the RBD from SARS-CoV-2 and seven animal sarbecoviruses was shown to elicit broad immunity in preclinical studies (160). Several unanswered questions remain around the clinical development of pan-sarbecovirus vaccines, including the type of immunogenicity data that will be required in addition to vaccine efficacy and the correlates of immunity that can be used (particularly for cell-mediated immunogenicity studies). It remains to be seen whether such approaches will result in improved breadth and duration of protection compared with current bivalent COVID-19 vaccines.

## Conclusions

Since the emergence of BA.1 in late 2021, new Omicron sub-lineages have continued to arise, superseding the previous Omicron sub-lineage approximately every 3 months. Later sub-lineages have had enhanced immuno-evasive properties and higher reproductive rates. Omicron sub-lineages continue to cause a substantial healthcare burden due to increased transmissibility, leading to a high prevalence of disease, and relative evasion of immunity, leading to re-infection and reduced VE. Novel vaccine strategies, such as variant-adapted vaccines or next-generation T-cell- enhanced approaches, are required to increase overall protection and durability of protection against symptomatic and severe infections caused by current and future Omicron sub-lineages, as well as other VOCs emerging from previously circulating variants.

## Author contributions

All authors contributed to the manuscript conception, writing, and review process, and approved the final version for submission.
