# Corrigendum: SARS-CoV-2 Omicron variants: burden of disease, impact on vaccine effectiveness and need for variant-adapted vaccines

**DOI:** 10.3389/fimmu.2023.1232965

**Published:** 2023-06-12

**Authors:** Shanti Pather, Shabir A. Madhi, Benjamin J. Cowling, Paul Moss, Jeremy P. Kamil, Sandra Ciesek, Alexander Muik, Özlem Türeci

**Affiliations:** ^1^ BioNTech, Mainz, Germany; ^2^ South African Medical Research Council Vaccines and Infectious Diseases Analytics Research Unit, Faculty of Health Sciences, University of the Witwatersrand, Johannesburg, South Africa; ^3^ School of Public Health, The University of Hong Kong, Hong Kong, Hong Kong SAR, China; ^4^ Institute of Immunology and Immunotherapy, University of Birmingham, Birmingham, United Kingdom; ^5^ Department of Microbiology and Immunology, Louisiana State University Health Sciences Center Shreveport, Shreveport, LA, United States; ^6^ Institute for Medical Virology, University Hospital Frankfurt, Goethe University Frankfurt, Frankfurt, Germany

**Keywords:** sub-lineage, BA.1, vaccine, disease burden, Omicron

In the published article, there was an error in the legend for **Figure 2** as published. The figure legend was displayed as “Favored cell entry pathways of (A) Delta variant and (B) BA.1. Delta favors cell surface fusion, whereas BA.1 favors endosomal entry. Evidence suggests that BA.4 and BA.5 sub-lineages may be partially reverting back towards cell surface fusion, due to increased fusogenicity compared with BA.1. Adapted from Tang et al. Antiviral Res (2020); 178:104792 (32)”. Furthermore, The figure legend included incorrect spelling of Cathepsin L as Cathespin L. The corrected legend appears below.

**Figure 2:**
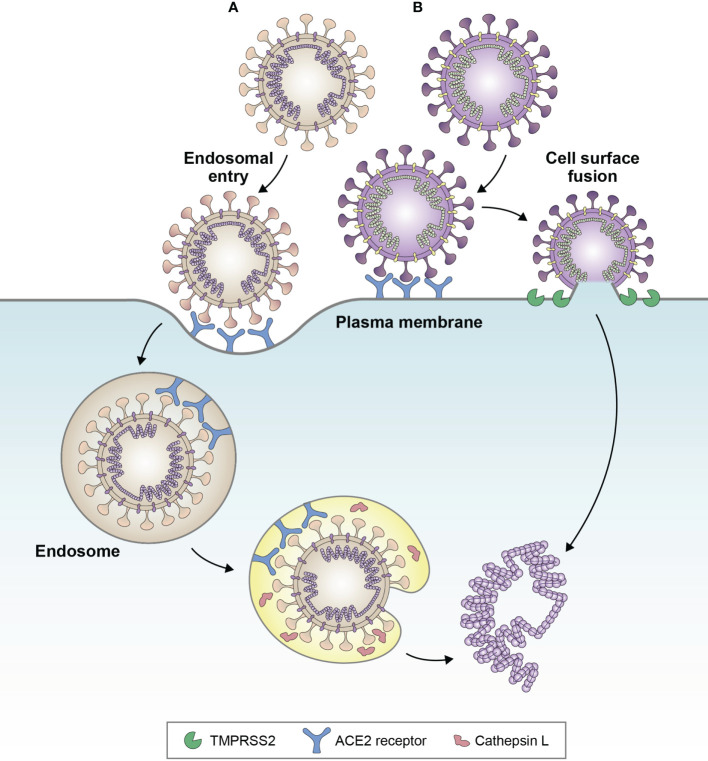
Favored cell entry pathways of **(A)** BA.1 and **(B)** Delta variant. Delta favors cell surface fusion, whereas BA.1 favors endosomal entry. Evidence suggests that BA.4 and BA.5 sub-lineages may be partially reverting back towards cell surface fusion, due to increased fusogenicity compared with BA.1. Adapted from Tang et al. Antiviral Res (2020);178:104792 (32).

“Favored cell entry pathways of (A) BA.1 and (B) Delta variant. Delta favors cell surface fusion, whereas BA.1 favors endosomal entry. Evidence suggests that BA.4 and BA.5 sublineages may be partially reverting back towards cell surface fusion, due to increased fusogenicity compared with BA.1. Adapted from Tang et al. Antiviral Res (2020); 178:104792 (32).”

The authors apologize for this error and state that this does not change the scientific conclusions of the article in any way. The original article has been updated.

